# Paper 1: Demand-driven rapid reviews for health policy and systems decision-making: lessons from Lebanon, Ethiopia, and South Africa on researchers and policymakers’ experiences

**DOI:** 10.1186/s13643-022-02021-3

**Published:** 2022-07-30

**Authors:** Rhona M. Mijumbi-Deve, Ismael Kawooya, Edward Kayongo, Rose Izizinga, Hadis Mamuye, Krystle Amog, Etienne V. Langlois

**Affiliations:** 1 The Centre for Rapid Evidence Synthesis, College of Health Sciences Makerere University, 1st Floor, Clinical Research Building, Mulago Hill Road, Kampala, Uganda; 2 The Regional East African Health Policy Initiative, Uganda country-node, Makerere University, Makerere, Uganda; 3 Liverpool School of Tropical Medicine, Liverpool, UK; 4 Ethiopian Public Health Institute, Addis Ababa, Ethiopia; 5 Li Ka Shing Knowledge Institute, St. Michael’s Hospital, 209 Victoria Street, East Building, Toronto, Ontario M5B 1W8 Canada; 6 Partnership for Maternal, Newborn & Child Health (PMNCH), World Health Organization (WHO), Geneva, Switzerland

**Keywords:** Rapid review, Rapid response services, Health policy and systems research, Universal health coverage, LMICs

## Abstract

**Background:**

Rapid reviews have emerged as an approach to provide contextualized evidence in a timely and efficient manner. Three rapid review centers were established in Ethiopia, Lebanon, and South Africa through the Alliance for Health Policy and Systems Research, World Health Organization, to stimulate demand, engage policymakers, and produce rapid reviews to support health policy and systems decision-making. This study aimed to assess the experiences of researchers and policymakers engaged in producing and using rapid reviews for health systems strengthening and decisions towards universal health coverage (UHC).

**Methods:**

Using a case study approach with qualitative research methods, experienced researchers conducted semi-structured interviews with respondents from each center (*n* = 16). The topics covered included the process and experience of establishing the centers, stimulating demand for rapid reviews, collaborating between researchers and policymakers, and disseminating and using rapid reviews for health policies and interventions and the potential for sustaining and institutionalizing the services. Data were analyzed using thematic analysis.

**Results:**

Major themes interacted and contributed to shape the experiences of stakeholders of the rapid review centers, including the following: organizational structural arrangements of the centers, management of their processes as input factors, and the rapid reviews as the immediate policy-relevant outputs. The engagement process and the rapid review products contributed to a final theme of impact of the rapid review centers in relation to the uptake of evidence for policy and systems decision-making.

**Conclusions:**

The experiences of policymakers and researchers of the rapid review centers determined the uptake of evidence. The findings of this study can inform policymakers, health system managers, and researchers on best practices for demanding, developing and using rapid reviews to support decision- and policymaking, and implementing the universal healthcare coverage agenda.

## Background

Globally, there are growing efforts to use evidence informed decision-making (EIDM) to address complex health questions. The World Health Organization (WHO)’s Strategy on Health Policy and Systems Research, *Changing Mindsets* [1], has advocated for further investment in this critical research area and greater generation and use of research evidence in health policy and systems decision-making [2]. Health systems worldwide often require contextualized evidence and within a limited timeframe to support pressing decisions for health systems strengthening [3, 4]. This has introduced rapid reviews as a useful approach to provide actionable and relevant evidence in a timely and cost-effective manner [5].

Rapid reviews respond directly to questions from policymakers and health system decision-makers. There remains, however, a significant gap in knowledge related to the conduct, contextualization, and use of rapid reviews in practical settings. Additionally, the process and experience of conducting rapid reviews from the perspective of researchers and policymakers, especially in low-income settings, have not been well documented.

To fill this gap, the WHO Alliance for Health Policy and Systems Research (hereinafter referred to as the Alliance) commissioned three institutions in low- and middle-income countries (LMICs) to pilot rapid review centers in 2016 and 2017. These three review centers were the South African Initiative for Systematic Reviews on Health Policies and Systems at the SAMRC (SAI; South Africa), the Centre for Systematic Reviews on Health Policy and Systems Research (Lebanon), and the Ethiopian Evidence-Based Health Care Centre, Jimma University (Ethiopia). Researchers from the Centre for Rapid Evidence Synthesis at Makerere University in Uganda acted as the technical assistance team and documented and evaluated the processes and experiences of the researchers and policymakers involved in the execution of the rapid response services (RRSs). The aim of this study was to appraise the experiences of these review centers in producing rapid reviews and using findings to enhance evidence-based decision-making for health policy and systems.

## Methods

### Study design

This was a case study employing qualitative research methods. A team of three experienced researchers in epidemiology and global health from the Centre for Rapid Evidence Synthesis at Makerere University in Uganda completed the data collection. The researchers purposively invited 30 people including researchers and policymakers who were identified by the centers as being intimately involved in the production and uptake of rapid reviews as either a researcher or policymaker (11 from Ethiopia, 14 from South Africa, and five from Lebanon). All identified individuals were initially contacted via email to invite them to participate in the interviews, and follow-up calls were conducted if needed.

### Data collection

The researchers conducted semi-structured interviews with the respondents using a research guide with open-ended questions that underwent pilot testing prior to the interview with the research team and policymakers in Uganda. Individual interviews were mostly carried out at the interviewees’ workplaces or over the phone, lasting about 45 to 60 min each. The researchers intended to explore the process and experience of establishing the centers, requesting a rapid review, collaboration between researchers and policymakers, disseminating and using rapid reviews for decision-making, and potential for sustaining and institutionalizing the services. All interviews were recorded following consent from the interviewees, and field notes were kept by the interviewers.

### Data analysis

Data from the recorded audio files were transcribed and analyzed using thematic analysis using NVivo 10 by QSR international. Two people manually and independently coded the data using inductive coding and organized the data into broad categories based on rapid response service activities and program components. The codes were further categorized into sub-themes, and the themes describing the most important factors related to the experiences of policymakers and researchers were evaluated. Respondents’ quotes were used to support the codes with major themes clearly presented in the findings.

### Data management

Data from the interviews and the results were stored in a password-protected folder that was only accessible to members of the research team. The data collected was checked for completeness before the study team member left the center and/or country of collection. This work was approved by the WHO’s Ethics Review Committee (review number ERC.0003016).

## Results

**Fig. 1:**
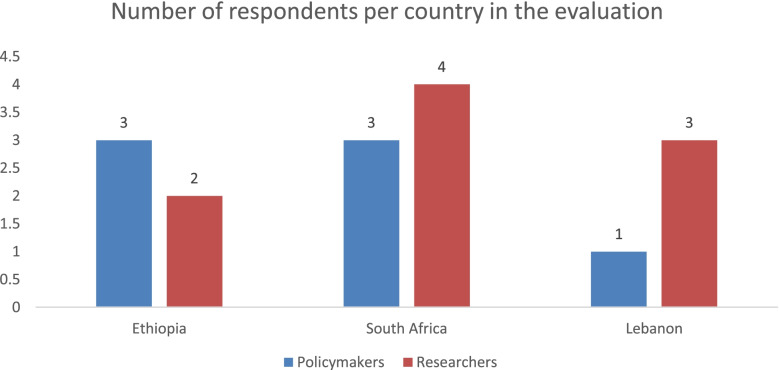
Profiles of respondents of the rapid review center evaluation in the three countries

Sixteen people agreed to participate, and their profiles are presented in Fig. 1. Fourteen people did not respond to the invitations or declined the interview after citing reasons such as not knowing enough about the review centers or that they were not permitted to speak on behalf of their workplaces.

Four major themes arose from the analysis, and together, they contributed to and shaped the final experiences of the stakeholders of the rapid review centers. The themes are presented in Fig. 1 and included the following:

i) Theme 1: Organizational structural arrangements
ii) Theme 2: Process management
iii) Theme 3: Rapid review products
iv) Theme 4: Influence/impact

Although these are represented as single entities, they intersection to produce the desired outputs. Organizational structural arrangements of the centers and the management of their processes are the input factors, while the products are the outputs. In turn, the engagement process and rapid review outputs contribute to the outcome of the rapid review centers around the uptake of evidence for policy and systems decision-making.

### Theme 1: Organizational structural arrangements

**Fig. 2:**
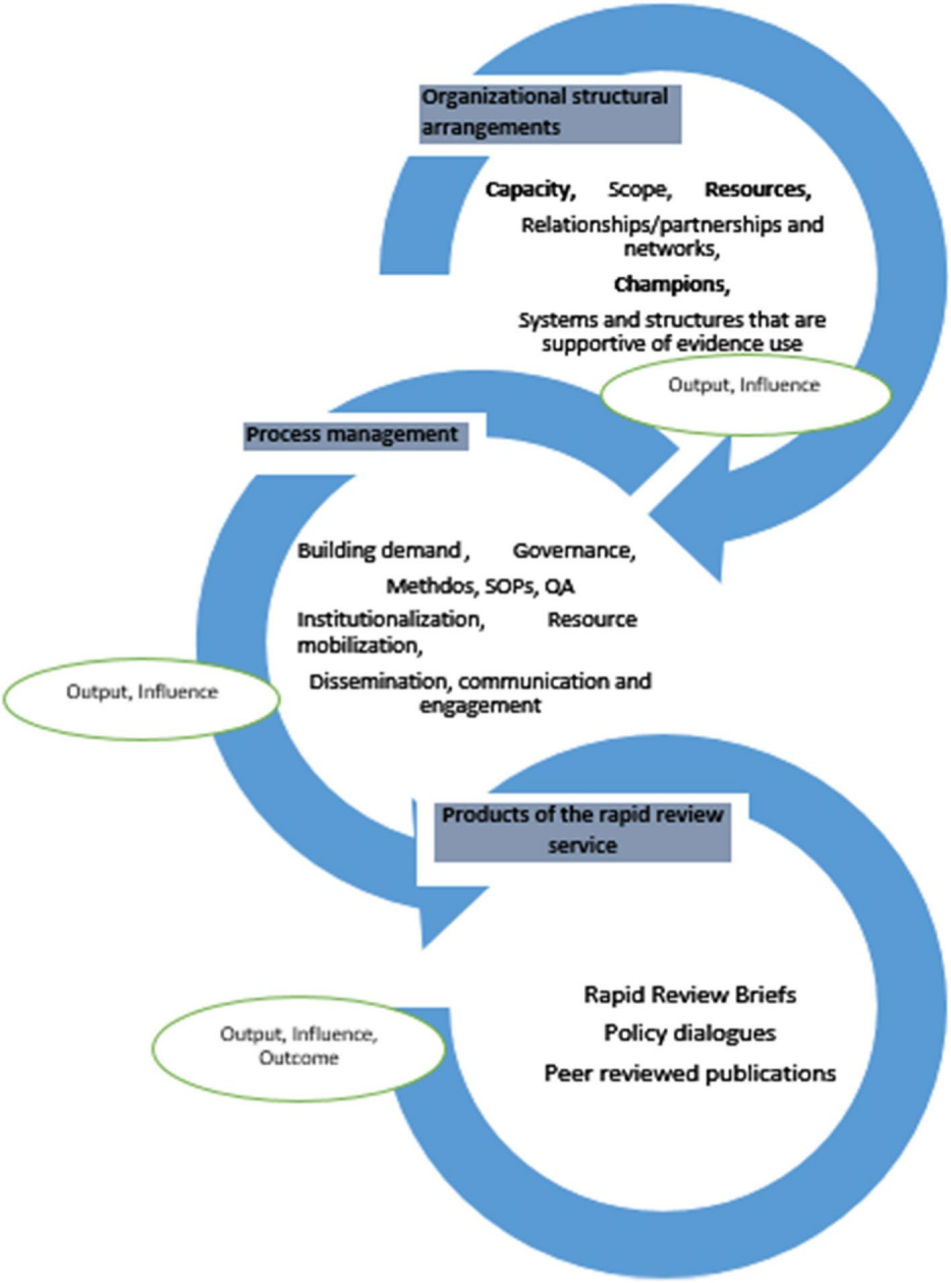
A figurative representation of the factors contributing to the experiences of researchers and policymakers of rapid review centers in Lebanon, South Africa, and Ethiopia

The formation of a structure is the initial step towards developing a rapid review center. First, it involves the need to identify the required capacities for researchers and policymakers. These capacities might include evidence synthesis methods, rapid review skills, and evidence-to-policy approaches and may be built iteratively. Development of these skills is incremental, contributing to the relationships and networks in building demand for policy-relevant reviews. As more demand-side human resources are established, more champions are made for the service, and the structure itself also increases visibility and awareness and gives credibility to the concept of EIDM (Fig. 2).

All selected countries were required to establish a structure for their rapid review center. In Lebanon, a pilot rapid response center was already in place; therefore, they consolidated and improved on the existing structure using information received from the technical assistance team. In Ethiopia and South Africa, there were no dedicated rapid review center at project onset. This meant that both countries needed to build an organized structure, which involved developing a team, mobilizing and ensuring resources, building vital relationships, defining the scope of the work to be done, and thinking about how to institutionalize the work that had begun.

#### Scope of reviews

**Fig. 3:**
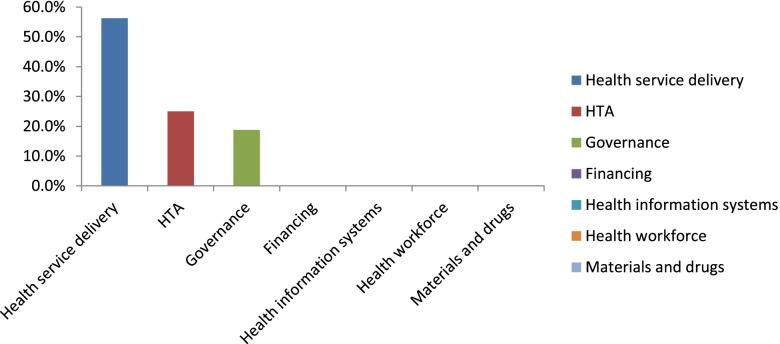
Types of questions received by EBHC from the Ministry of Health directorates during the pilot of the rapid review center

**Fig. 4:**
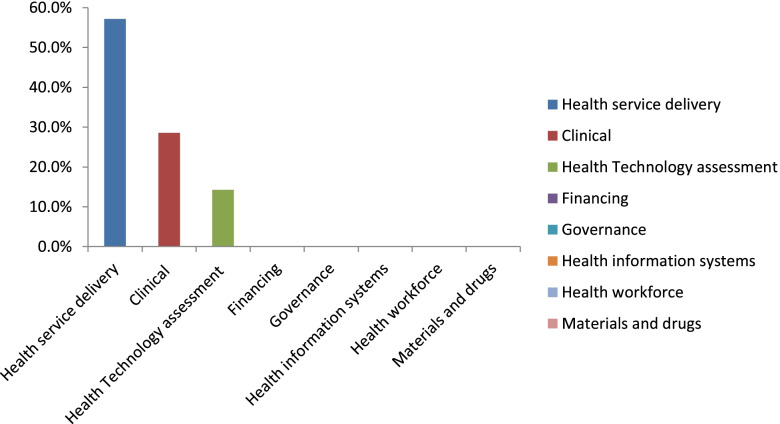
Type of questions received by SAMRC from the district Departments of Health during the pilot of the rapid review center

The teams realized the critical importance of defining the scope of the questions they could take on (Figs. 3 and 4). The scope was mostly determined by the teams’ capacity in terms of the number of team members and/or content experts available. For two of the three teams, the scope was defined iteratively, and for one team, the scope was pre-defined as they had already been involved in planning for rapid response work around universal health coverage even before the grant. However, all teams generally agreed that it was important for the scope to be clarified for the knowledge users in order to manage their expectations.

#### Strengthening capacities

On the onset, the centers recognized that there was limited capacity on the team for rapid reviews. As such, one of their first priorities was to build capacity for researchers and their advisors, policymakers, and institutions.

#### Supply side capacity — researchers

The centers were generally established within academic institutions that had little experience with structured activities around engaging and responding to the policymaking processes. Only the institution in Lebanon had carried out related activities but not in the context of rapid reviews. Therefore, it was crucial to build this capacity within these teams.

Most of the researchers were qualified and well trained, particularly in systematic review and rapid review methodologies, but they reported that they needed additional training to respond to the policymaking process, relevantly and comfortably. The respondents noted that the training should not be a single occurrence, as one session may not leave people feeling confident enough to continue on their own. Respondents also noted that additional support or mentorship was necessary to ensure that they applied what they learned into practice. Given this, it was important for all the teams to have access to training and make appropriate choices about who to invite to the trainings. The South African center included people from several collaborative institutions in addition to their internal team, while the centers in Ethiopia and Lebanon focused on groups within one organization. The South African group’s approach allowed them to continually draw on people from outside of their immediate team for different parts of the rapid response process. One of their researcher’s said: “The people who came for [mentions name]’s training we involve them like whenever we have a question. We put it out on to this mailing list of people who came for training and those who are interested in that topic volunteer to work on it.” It is rare that these capacities will be seen in one individual, and so, one may look to formulate a team with several individuals with one or some of the desired knowledge and skills. The policymakers also mentioned the need to set up structures to support use of evidence and having skilled individuals within the ministry who will be able synthesize and translate the evidence provided.

 …………there needs to be [specialized capacity building] within the team to be able to do these rapid reviews succinctly but also to do it well. …………. There is need for capacity to be able to write well and to package it well…………. You need dedicated capacity to search efficiently to identify evidence and then to be able to go through the evidence and be able to make sense of it. (Researcher, Ethiopia)

In addition, the teams realized that there were other capacities outside of the traditional technical ones that were needed. These included the capacity to engage with, negotiate review questions, and understand the policymaking process, as described by a policymaker from Ethiopia: “And I think the other important thing is to understand the decision makers’ world because if you are a researcher you don’t always understand what is on pressure and the issues on the policymakers’ side.”

#### Dedicated capacity for the service

To be efficient, respondents noted that there must be dedicated capacity for this kind of work. Several respondents from both the supply and demand side felt that this was critical for the survival of such a service:

So I think for me the critical thing about these kinds of projects going forward is that it needs dedicated capacity. It can’t be something that somebody adds on in the day and do it for the last or after hours for instance. You need dedicated people that can foster the relationship. That can even be in the policy maker space to understand the question that is coming up (Researcher, South Africa).

### Demand-side capacity — policymakers

There was general consensus amongst the respondents that the actors in the policymaking arena needed a certain set of knowledge and skills (e.g., reading and understanding research or synthesizing evidence) to be able to use the rapid review findings effectively and efficiently. It was also beneficial for policymakers to attend the review centers’ trainings as they developed a better understanding and level of engagement with the process.

……………She [a policymaker] was very specific and clear and I think it is because: one; she has got a background in training in research and two; she came for the training with [mentions name] so she sort of understood what a rapid synthesis is and can provide. Whereas with [mentions name and background] and there is [mentions name and background], I think they are both …………..I am not sure how much of research background they have because we struggled a lot with moving their questions from operational to reviewable questions (Researcher, South Africa).

When the participants were asked about what the needs of the policymakers were, one policymaker emphasized the need for them to also be capacitated to make use of the service. She noted, “It is how to make use of the information because you can generate a whole lot of questions but how do you use the information that was generated” (Policymaker, South Africa). Another policymaker highlighted that one of the barriers to the rapid review services is “our capacity in interpreting the response report,” which needs to be addressed to ensure that the center is successful.

#### Efficiency

A policymaker from Ethiopia, who had been using evidence in the form of surveys for decision-making, felt that the rapid reviews produced by the centers were less resource consuming in terms of time and funds as compared to the alternative sources.

#### The evidence (availability, relevance, timeliness)

Participants felt that the determining factor for a good experience with the service was the evidence itself in terms of availability, relevance, and timeliness. Overall, the respondents felt that the rapid review centers were able to provide policy-relevant evidence in this way.

#### Relationships, partnerships, and networks

Partnerships and collaborations were credited for different strengths brought to the process. These contributions included improved capacity, strengthened methods, and improved demand.

A researcher from South Africa noted that collaboration strengthened the methods and the guidance to be able to implement the project. Specifically, they were drawing on several people from partner organizations outside of their group who participated in the training. Respondents also mentioned that the relationships between the researchers and policymakers were important for RRS work. They pointed out that several factors were involved in building and maintaining these relationships, particularly trust and credibility.

A participant stated that it was important for expectations and responsibilities of each party to be clearly outlined:

I think if you have a clear terms of reference in the agreement as to what from both sides. What is your role and responsibilities as the policy maker and what is your role and responsibility as the service provider? ………………. it will serve to guide to say this is how we will work together.

#### Stakeholders and media

Other relationships were highlighted as important. For example, one policymaker from Ethiopia noted that it was important to involve the media in understanding rapid review work as they would in turn foster linkages between the researchers and policymakers. He said:

If we train for example the mass media about evidence synthesis . . . I think the mass media could play a pivotal role by linking the researcher, the evidence synthesis [team] with the policy makers. And if the mass media is provided with this synthesized evidence, they can make it public and they can go to the policy makers to also with this evidence and ask the policy makers whether they have used it or not. Or the other way around [to the researchers], saying the policy makers and decision maker are noting this evidence (Policymaker, Ethiopia).

#### Champions

Policymakers frequently act as champions spreading the word amongst their colleagues and other potential users. For example, a South African policymaker from the State Department of Health who used the service mentioned that he heard about the RRS “From [name], my colleague who attended a training.” A researcher from Ethiopia also commented on champions: “So otherwise, the methods are very clear. Once we get a champion within the ministry of health we will establish that place and strengthen that relationship and we will push that structure is also created at the ministry of health.”

#### Institutionalization

All respondents agreed that the activities involved in the RRS should be institutionalized and made more permanent and sustainable. A policymaker from Ethiopia emphasized the following:

……………….if it has to be sustainable the way forward is through permanent structures not committees. And advisory councils get to advise not to synthesize evidence. We have made it in black and white for [the ministry]. …………………….. But for the policy makers around the ministry of health, it’s always the council. Because of that it isn’t working [very well yet].

The respondents suggested that skilled staff and permanent platforms for rapid review centers were primarily needed for sustainability. However, they argued that it was important to determine where and how the service added value to each institutional level.

…………………. so the one [level] we are currently at is what we call the middle level. And we are in the strategic unit so we are responsible for the planning and designing of service delivery. But then we also have a macrostructure which is responsible for policy development . . . So for me I find it valuable because we need to take policy and make it operational and implement it . . . so we look at what is the evidence based on feasibility, sustainability looking at making things work within your specific context. …………..at National department of Health level they are responsible for setting policy………………. So at that level it would make sense to have access to such a service because by the time it comes down to us they should have gone through all those thinking processes (Policymaker, South Africa)

### Theme 2: Process management

In order for the structure to be functional, processes and their management must be in place that support policy and systems decision-making. As the centers carry out activities to build demand, they continuously engage with policymakers, which leads to increased demand and knowledge of policy needs and contributes to institutionalization.

#### Building demand

Rapid review services are ideally demand driven and aim to respond to the concerns and queries of the policymaking process. Therefore, research questions are typically generated by policy and other decision-makers and presented to the review team. To facilitate this process, the researchers from the RRS must build a relationship with the policymakers based on trust and credibility. One center noted, “It may take time, it’s like change of culture. Cultures don’t change overnight.”

To begin building new bridges, one center described how they leveraged their existing relationships: “Other relationships we already have, [we] tried to use them to get more people to seek our service.” Another method of stimulating demand is to invite policymakers to rapid review training. Respondents also reported that researchers needed to leverage the results from completed reviews to gain credibility. For example, a researcher from Lebanon stated: “I think for a time being the researcher should go and show them that these are the results that show that evidence can be synthesized within a month or so. The advantages of decisions based on evidence is more higher than decision without any evidence.”

#### Leadership (who is in charge and who owns the process)

The issue of process responsibility arose. The policymakers submitted their queries and utilized the final information, but the researchers conducted the reviews and synthesized the data to respond to the research question. Therefore, some clarity was needed around who held the responsibility to lead and see the knowledge translation (KT) process through. The responsible party — supply or demand side — would need to be well equipped to manage the process and would be liable if it did not evolve appropriately. This would also assist in evaluating and improving processes.

#### Methods, standard operating procedures, and quality assurance (ethical issues, management of the evidence synthesis process)

Developing methods and standard operating procedures (SOPs) is an iterative process that is supported by the growing experience of the team. For example, teams realized that continuous engagement with the policymakers was required and should be deliberately planned for with clear objectives. A respondent noted:

The first person we engaged with ……………… the objective was to actually conduct the full rapid synthesis review in two weeks……….he gave us the question ……….. but when we read about it we realized that there were missing parts and there were things which we did not understand so we went back to him. But at that point we [had] not seen us going back and forth as part of the whole package, it was [originally] like all just a minor thing to help us……… (Researcher, South Africa)

Centers recognized that the RRS model, which was presented to them during the trainings, needed to be adapted to their own contexts and capacities. One researcher indicated, “We actually changed our model from trying to come up with the 15 days finished product to actually starting small rapid responses” (Researcher, South Africa).

#### Negotiating and clarifying the review question

Both the researchers and policymakers were in agreement that the initial clarification process needed to be standardized to ensure that the needs of the policymakers and the research questions were well understood. A policymaker pointed out:

The kind of questions we were asking are practical questions and it is important that the researchers understand the health system’s workings. Clarifying the question took time; we kept coming back and forth. It was good that they brought in an expert to clarify the question (Policymaker, South Africa)

The clarification process may take time, but it is an important step, and as the policymaker noted, the review team should have sufficient health systems’ knowledge and skills.

#### Engagement and communication

The review centers and policymakers faced challenges with engaging and communicating with each other. For example, a researcher recalled their initial attempts at communication with policymakers:

So the first meeting when we were negotiating the question we asked them which is the best form of communication and most have said emails. But we also understand that they are very busy people sometimes they do not respond at all and we might have to make a phone call to follow up. (Researcher, South Africa)

Referring to the same incident, a policymaker said, “We had some meetings and used email but you know emails can get lost in the day to day mails that come in so it took some time” (Policymaker, South Africa). Furthermore, policymakers suggested that the researchers meet with them and interact with the policymaking processes to determine needs and learn more about how each side works. Respondents highlighted that time was a big factor in this kind of engagement. Both parties were quite busy as they were usually engaged in several activities in addition to their day-to-day work. It was important that both sides appreciated this right from the start. Continuous engagement on any given piece of work was also identified as one way of getting both groups to understand and contribute fully to the RRS processes. In Ethiopia, a co-production model was used when addressing a particular policy question through a rapid review. Policymakers worked alongside the research team to produce the findings. One respondent described their experience: “From our side we assigned focus for each research question and from their side they assigned like a Principal Investigator on this research question. And they communicate with the focus and the PI. They need like some document some guidelines from our side. They call, they email from the focus, from the PI”(sic) (Policymaker, Ethiopia).

#### Human resources

All participants agreed that the supply side human resource (i.e., the researchers or brokers of knowledge on the teams) were vital to the review center’s success. These resources needed to be mobilized in terms of the number of team members and their time commitment to the RRS. All of the centers in this study utilized researchers who were working on the projects part time or in addition to their usual work, but one researcher highlighted that these individuals would have to be incentivized (e.g., financial, academic, or professional) to ensure efficiency. Such incentives could include the following:

… providing additional towards the salary, additional money could be helpful. We have put for example in our proposal we have put some incentives as an increase to the budget. And the other is for example the rapid review products should be used for promotion within the institute. And they have to be properly trained, training by itself could be considered as an incentive on people get new skills when they get say confidence. So training them, providing them with some additional incentives, money and considering the products for promotion at the institute (Researcher, Ethiopia).

### Theme 3: Rapid review products

#### The products

The rapid review products generated by these rapid review centers were secondary outputs of existing research and evidence. The policymakers felt that this might be a limitation, and that the centers could consider producing more forms of evidence:

Yeah so actually one thing that I mentioned previously using the routine information system as one way of like triangulating the findings that maybe good….it’s good if they use the routine data. In fact it is rapid but sometimes it may be good if they use like primary source of data. They use like research. The rapid actually, yeah. They can add some types of qualitative tools probably like interviewing with programs people, one or two program people and then they can add onto their report. (Policymaker, Ethiopia)

#### Use of the products

The researchers indicated that the products and the evidence they provided were generally used in the decision-making process. One researcher said:

But then when we went to talk to [mentions name] about her question what we found out is that actually the information which we had gathered and given …was actually used as part of the decision making. And just a few days ago we talked to [mentions name],……..he also said yeah the information which we did give to [mentions name] was part of the decision making. We are not sure how much it influenced the decision but it actually made a difference [for us]. So I think there is a part for the service in policy making. We just need to find out how much is actually used. (Researcher, Lebanon)

Knowing this, the researchers were motivated to continue with the rapid review work despite feeling that the process was difficult.

### Theme 4: Influence/impact

#### Influence on knowledge, attitudes, and practices

Both the researchers and the policymakers noted that there were changes in knowledge, attitudes, and practices during their engagement with the rapid review centers. In this case, the researchers reported how the process helped them gain clarity on the different rapid synthesis processes and products. For example, several researchers and academics noted that there was enhanced clarity from the more academic rapid reviews, that they were more conversant with and could relate to better, towards the user-side oriented rapid responses, and that there were more than just the synthesis but were an outcome of an interaction process and engagement with the user side. “So it has changed from the first rapid synthesis we had. And I think also with that said it’s not an official name change but we have started calling it more of a rapid response as opposed to [just a rapid review]” (Researcher, South Africa).

The researchers and policymakers also gained a better understanding of health systems and their application and different components related to the rapid review work: “So I know the difference between health systems and epidemiology but until I started working here it was more theory based (the health systems part)” (Researcher, South Africa).

#### Research prioritization

The review center teams found that the results of their research fed back into the process of research generation. For example, researchers in South Africa reported that the evidence from the reviews was often missing localized data. As such, they worked towards filling in those gaps with the available opportunities and partnerships. One researcher from South Africa reported:

…….. when we find gaps like when we are looking at what [mentions name] really wanted; …………..there was no study at all which was done in South Africa and so what then? So now we know and what do we do with that information? So how do we go a step further may be working with UWCT and UCT to actually use our findings to the stuff which masters students or PhD students can fill the gap so that it becomes the whole around system…….. because I know that the department of health they are trying to work more with Universities to create research which is relevant to their questions. So we can try to fit into that.

The policymakers also recognized the gaps in evidence and the need to fill them. One policymaker suggested:

I think there is a key role in if they find for example when they find very little evidence of or very little reviews if I can go to the systematic reviews out there. It’s to feed that information back to your research institutes to say this is a gap in evidence that we found. Possibly these are the type of questions that has been asked. Start directly employing more role in agenda setting for primary researcher as well (Policymaker, South Africa).

### Policymakers’ perceptions

Despite facing some challenges throughout the pilot period, the review centers were successfully established and were able to conduct demand-driven rapid reviews. One method of measuring success is user satisfaction or appreciation. In general, policymakers were pleased with the review centers’ responsiveness and final products. In South Africa, for example, a policymaker described the deliverables as being of “good quality” and completed relatively timely:

On our part we were constrained by time, the SAMRC …..we received a report from them which was good quality…... Timeliness…on the first question, it was a bit slow but it was understandable since this was a first time for them, but time for the second question was good…it took about 2 weeks (Policymaker, South Africa)

Another policymaker noted that the provided evidence enabled their unit to know and understand the gaps in evidence, which was important for determining how they would execute their proposed program. This also applied to situations in which no evidence was found to answer the research question directly. One policymaker reported:

We were satisfied with the quality of the work. There is no complaints related to that and unfortunately the work we have asked there wasn’t a lot of primary studies and a whole lot of synthesis already being done on this. So a no answer is also a good answer . …. if a policy maker does not have a good research understanding, they have to understand that your answer that you are getting is also an answer. It is not a reflection of a poor outcome or a poor result ….. (Policymaker, Ethiopia)

The policymakers also described some limitations to the RRS and provided suggestions for improvement during their interaction with the researchers. For example, the policymakers felt the RRS should have a wider scope and not just focus on health system questions. The policymakers appreciated the value of the rapid response service and focus on the questions but suggested that the summaries add recommendations for them to act on.

Of course so, these are some of the benefits. That we got from them and they give like some 2 or 3 recommendations from policy makers like us. Yeah, it’s you know doable just to give 2 or 3 recommendation areas. That this is you know I don’t think that we reached on matched states (Policymaker, Ethiopia).

#### Opportunities

All surveyed policymakers agreed that there were many instances where they needed evidence in an urgent manner, and they felt that the rapid review centers provided an appropriate, timely, and cost-effective avenue for them to access evidence in these situations.

Yeah………… I feel that that’s a very good way of like getting information for decision. Because when I say survey - survey is good but we have two problems with survey. The first thing is survey is resource intensive. You need to have huge resource ………… to invest millions of pounds or dollars.………….. The other problem is time because these surveys take like a year or more than a year or something like that. So, if you are in urgency or if you need an urgent decision this survey may not be the way. (Policymaker, Ethiopia)

Another policymaker felt that there were many opportunities in the policymaking cycle in which the RRSs could be useful. She emphasized the need for the researchers to stay in touch and connected with the policy process. For example, there are political windows when specific issues are addressed and are on the agenda. The researchers need to be more involved to get to know these opportunities and be able to provide support when it is relevant.

#### Challenges/threats

Although there was satisfaction registered amongst the policymakers, there were some challenges identified by both the researchers and policymakers. “Yes, I would use the services but rapid response should be quicker, policy many times needs quick responses” (Policymaker, South Africa). The researchers felt that some of the activities involved in the rapid review process were onerous and could be streamlined or eliminated, “So I think our biggest thing right now is finding ways for people to seek the product without us having to do all the hard work of knocking on their doors. I think that would be really great for our sustainability” (Researcher, South Africa). In addition, researchers found it challenging to reconcile their knowledge and definition of good evidence with the needs of the decision and policy-making processes that may not necessarily align with that knowledge:

MSF came up with the idea - they came up with adherence clubs for HIV positive patients. So they are not a research centre they do not go through whole what research would say is rigorous and so most about 70 percent of the information which we getting was through MSF, through funders’ reports ……….. And so if you are in a research institute that might not be seen as rigorous but it might be the only information which exists. So do you then say there is no information or do you actually include that? (Researcher, Ethiopia).

Both the researchers and policymakers were faced with the issue of high staff turnover in the ministries. Since the majority of the rapid response work was dependent on building long-term relationships with policymakers to facilitate behavior change, the staff turnover impeded on this process. In Ethiopia’s case, this issue threatened to derail the RRSs altogether.

So I think the challenge is staff turnover. That’s the major challenge we have as researchers and policy makers especially the policy makers. For example last time there was this guy who was a secretary of health systems research advisory council and I was a chairperson of that group, and when we were trying to come up with terms of reference (for the rapid review centre), the next meeting I couldn’t ….. because the secretary who was located at the ministry office had left the ministry (Policymaker, Ethiopia).

Resources and capacity were limitations mentioned by both researchers and policymakers. The review centers were not able to meet the high demand for the RRSs because of their limited capacity: “Probably the short comings are related to I don’t know probably the financial capacity. Because we received like more than 20 questions but we select some yeah because of the limitation of the resources” (Researcher, Ethiopia).

#### Interaction of the four themes

Together, the organizational structural arrangements, the management of processes, and the products of the RRS shaped the outcome and/or influence and/or impact of the RRS. For example, the interaction and engagement of these components allowed the demand side to understand the needs of policymakers, while the policymakers understood the benefits and modalities of how to work with evidence. Additionally, feedback loops may develop between the structure and process management. The continuous engagement between these two mechanisms means that whatever is learned in the process is fed back into the structures. This leads to the creation of a built-in check system that ensures that the system is continually improving, learning from itself, and correcting the gaps.

## Discussion

Our study highlights that policymakers welcome the idea of being engaged in platforms and strategies to ease the reported barriers that they face as they attempt to use evidence to inform policies, decisions, and practice. Researchers also benefit from similar strategies for the purposes of understanding, accessing, and engaging with the policy arena. As such, knowing about the experiences of both sides stimulates attitudes towards the use of evidence for decision-making in general.

Our research sought to appraise and understand the experiences of rapid review centers in Ethiopia, Lebanon, and South Africa in conducting and using review findings to support policy- and decision-making processes in their countries and to understand the issues around the nexus between rapid reviews and policymakers’ needs for evidence. A study exploring the views and KT experiences of 14 Australian public health academics found that the capacity to engage in KT was influenced by factors within the academic context and the interaction of the academic and policy environments [6]. We expected to find that the experiences of researchers and policymakers would be similarly shaped.

Cherney and colleagues [7] described several factors that influence the utilization of social science research (i.e., contribute to one’s experience). These factors can be grouped into four broad headings, and these align with the themes in our study related to the context of researcher, end user’s perception, dissemination, and interaction between the researchers and end users. Studies have suggested alternative groupings for the same factors, which include individual or person-specific factors, and organizational level factors [8]; internal, external, and environmental factors [9]; or factors external to the policy organization such as stakeholder and government views and perspectives [10]. The factors in these studies, although categorized differently, are generally similar. For the purposes of our evaluation, we found it was most appropriate for the factors influencing the experience of users and researchers on the rapid review centers to be categorized by organizational structural arrangements, process management, and factors related to the end products, all of which culminate into a form of influence or impact on decision-making. Organizational structural arrangements include scope of reviews, strengthening capacity, the availability, relevance and timeliness of evidence, relationships, partnerships and networks, champions, and institutionalization. Process management includes leadership, methods, engagement and communication, and human resources. Rapid review products include the products and use of products, and finally, impact includes influence on knowledge, attitudes and practices, research prioritization, policymakers perceptions, opportunities, and challenges or threats.

A departing point in our study is the interaction of themes and emergence of the outcome and influence category, which was seen as an endpoint, but may also be a factor that influences or contributes to the endpoint. This highlights that there are often no single endpoints (e.g., products being used in practice) or a well-defined one in processes related to KT. Indeed, the process involves all kinds of activities between generation and use of the evidence, which should culminate into better use of evidence. In many cases, this is thought of as a single process ending in use, but this may not be the case. For example, the influence of engagement during the rapid review process may result in a change of attitudes or incite curiosity in a policymaker who may use evidence as part of another process. The same policymaker could later act as a champion, leading peers and colleagues into considering EIDM. This endpoint is a byproduct of the main process and does not necessarily happen at the defined end.

In this study, it is also necessary to understand the fact that rapid reviews and systematic reviews are still largely viewed as an academic exercise. Therefore, it is not surprising that researchers were still struggling with issues around rigor and quality and methods of knowledge syntheses as may be required to support policymaking. Many studies have documented that rapid reviews and knowledge syntheses in general are not well accepted by academic peers particularly in the policymaking domain. This could be a result of the absence of an agreed methodology for conducting rapid reviews to support policymaking or the fact that rapid reviews are perceived as inferior to other traditional research methods [11]. Contrary to this viewpoint, evidence suggests that rapid reviews may be advantageous in the policy process. For example, rapid reviews are context and organization specific [12], the methods are seen as “flexible and pragmatic” with the aim to balance the objectivity and rigor required of rapid reviews within a limited time frame [13], and they improve clarity and accessibility of research evidence for decision-makers [11]. The benefits of accessibility were particularly evident in the study based on the feedback received from policymakers. They expressed that they had easier experiences with the RRS because they were able to access evidence in ways they normally would not have. For example, a policymaker mentioned that carrying out surveys to answer every research question was costly in terms of time in comparison with having a rapid review prepared.

A recurring and important factor that determines the experience of both researchers and end users is the capacity on both sides to interact and make sense of the rapid review process. On the researchers’ side, the capacity to work beyond the academics of the review and interpret or contextualize the review questions with the policy process in mind calls for particular skills that they may not have on the onset. If these skills are not developed, there is a potential for the review centers to fail to progress, which the respondents in this research highlighted very well. Researchers at the SAI reported having minimal experience interacting with policymakers, and on one occasion, this affected the demand or willingness for a policymaker to accept the RRS that was offered. All models of knowledge transfer recognize that KT of evidence especially research findings requires active engagement between researchers and users. However, this is often also cited as a barrier because of the two communities running on parallel agendas with minimal engagement or understanding of each other’s needs and norms [14–16]. Similarly, it is important that policymakers have a set of skills that enables them to interact meaningfully with a rapid review team or its products. Very often, the focus is centered on the capacity of the researchers or the supply side to provide relevant evidence to the policymakers with no emphasis on the policymakers’ responsibilities. In other places, policymakers highlighted the difficulties for these transfer processes to happen without their capacities matching up to the needs of the process and what their roles should be, including receiving or reaching out for the research, understanding it, and using it [17].

Mijumbi and colleagues have emphasized the need for resources to support KT activities to ensure that they are adequate and, most importantly, sustained [18]. However, the necessary funds and resources are not always available particularly in LMICs, as many of these services are not government funded, and they do not have established budget lines. The teams involved in this study received funding from the Alliance, but it was not clear how they planned to support their activities beyond the grant. This is an important issue for teams to address so as not to lose the gains made during these pilots. The capacity to conduct this kind of work from a human resources perspective is limited, which also poses an issue, and some incentivization is needed to ensure “retention” or sustained availability. In this study, nonfinancial incentives that speak to the profession or the career of the researchers (e.g., recognition of effort when preparing a review) were emphasized. The purely academically published work is recognized but not at the nexus of academia and practice or policy, at least not in a standardized way that would ensure career advancement. An analysis exploring organizational factors that influence university-based researchers’ engagement in knowledge transfer activities found that one of the barriers described in the literature was the reward and incentive system of academia. In general, this system continues to value traditional types of within-group activities including publications in peer-reviewed journals, presentations at disciplinary conferences, and receipts of research grants over the more broadly directed outreach and production activities associated with knowledge transfer [14]. Therefore, with this low value on KT activities and other competing and yet demanding academic activities, KT will be ranked low on most academic priorities, which in turn means few researchers will invest the time and resources in getting training and/or experience with KT.

As governments and their stakeholders including researchers and knowledge brokers push forward with the EIDM agenda, it is important that they all create an enabling environment for the practice. Knowing the factors that affect the experience of both researchers and users of evidence as presented by this study is helpful in ensuring this enabling environment, which would eventually contribute to uptake of evidence and a culture of EIDM. This study also highlights the importance of policymaking institutions ensuring that their staff and other stakeholders involved in the policy processes are well equipped to allow for efficient engagement and use of synthesized evidence. The same applies for the supply side, which includes research and knowledge-brokering institutions. Lastly, this research presents elements supporting the experience of producers and users of rapid reviews in response to policy and decision-making.

The strengths of this research include utilizing methods of implementation research, implementing an intervention in real-life settings, and evaluating and learning from it. This is one of very few studies on rapid review services preparing rapid reviews for policy- and decision-making in LMICs, thereby increasing our understanding of the field of EIDM in these settings. The main limitation of this study is that it is based on pilot rapid review center; therefore, it remains to be seen if the same results would be observed at scale. In addition, case studies are the main method used in this study, with inherent limitations pertaining to external validity and application in other LMIC settings. However, we find that case studies are appropriate for pilots of this nature given that the research is exploratory and we are attempting to generate an illustrative theory about the experiences of individuals [19].

## Conclusions

The experiences of policymakers and researchers during the setup of rapid review centers and the process of providing rapid review products determine the uptake of evidence. These factors interact at different stages to influence the impact of the rapid review centers and evidence. The findings of this study can inform policymakers, health system managers, and researchers on best practices for demanding, developing, and using rapid reviews to support decision- and policymaking and implementing the universal healthcare coverage agenda.

## Data Availability

The data is available on request to the corresponding author Rhona Mijumbi-Deve.

## Abbreviations

Alliance: Alliance for Health Policy and Systems Research
EIDM: Evidence-informed decision-making
KT: Knowledge translation
LMIC: Low- and middle-income country
RRS: Rapid review service
SAI: South African Initiative for Systematic reviews and Rapid Evidence Syntheses on Health Policies and Systems
WHO: World Health Organization
